# Coexistence of monogamy and polygyny in *Triatoma infestans*: fine-scale genealogical structure reveals complex social structures within domestic colonies in the Bolivian Chaco

**DOI:** 10.1590/0074-02760250355

**Published:** 2026-06-15

**Authors:** Alex Juan Cornejo Pinto, Rolando Sánchez, Frédéric Lardeux

**Affiliations:** 1 Pan American Health Organisation/World Health Organization, La Paz, Bolivia; 2 Universidad Mayor de San Andrés, Instituto de Biología Molecular, La Paz, Bolivia; 3 Institut de Recherche pour le Développement, France

**Keywords:** Chagas disease, Triatoma infestans, genetic structure, mating systems, vector control, Bolivia

## Abstract

**BACKGROUND:**

Persistent reinfestation of *Triatoma infestans* in the Gran Chaco undermines Chagas disease (CD) control. While insecticide resistance is a known factor, the fine-scale social and demographic structure of vector colonies remains poorly understood.

**OBJECTIVES:**

To analyse the micro-geographic genetic structure, mating systems, and demographic history of *T. infestans* in a rural community in the Bolivian Chaco.

**METHODS:**

We genotyped 381 individuals from three neighbouring structures (domiciliary and peridomestic) using eight highly polymorphic microsatellite loci (expected heterozygosity, *H*_E_ = 0.675). Genealogical relationships were reconstructed using maximum likelihood (ML) analysis (COLONY), and demographic history was inferred through heterozygosity tests.

**FINDINGS:**

The infestation comprised 42 distinct full-sibling families nested within a single colony. We observed a stark dichotomy: two dominant families (> 80 individuals) exhibited monogamy and signs of distinct demographic histories (one in expansion, one recovering from a bottleneck), while 40 minor families showed high promiscuity (polygyny/polyandry). Finite growth rates varied significantly (λ = 1.03 vs 1.22), evidencing active intra-colonial competition.

**MAIN CONCLUSIONS:**

Domestic populations are not random aggregates, but complex mosaics of coexisting lineages with different adaptive strategies. The dominance of monogamous families suggests a priority effect advantage, while the "tail" of promiscuous minor families represents a cryptic reservoir. Effective control must account for this structural resilience, as surviving minor lineages could rapidly recolonise the niche.

Chagas disease (CD), caused by *Trypanosoma cruzi*, remains a major public health challenge in Latin America.^1^ Among its vectors, *Triatoma infestans* (Klug, 1834) is the most significant species in the Southern Cone, particularly in the Gran Chaco ecoregion. The taxonomic classification and historical monitoring of these vectors rely heavily on foundational monographs, such as Lent and Wygodzinsky,^2^ which must now be integrated with modern perspectives on new triatomine species challenges and citizen surveillance strategies.^3^,^4^,^5^,^6^,^7^,^8^. Despite decades of coordinated control efforts and successful large-scale interventions — such as the Pampa del Indio project in Argentina, which achieved district-wide quasi-elimination^9^ — *T. infestans* remains a persistent threat in the Bolivian Chaco due to complex reinfestation dynamics.^10^

Standard control protocols typically treat the infested dwelling as a single epidemiological unit, assuming panmixia (random mating) among the localised vector population. However, theoretical models and limited field data suggest that vector populations may be structurally subdivided.^11^,^12^ If a domiciliary colony is a mosaic of competing families with distinct reproductive strategies and spatial behaviours, this could explain the resilience of infestation: a focal spray might eliminate the dominant lineage while leaving subordinate, cryptic families to recolonise the niche.

In recent years, the use of molecular tools, specifically microsatellite markers, has revolutionised our understanding of vector population dynamics.^13^ However, the internal social and reproductive structure of these colonies remains poorly understood. While polyandry (females mating with multiple males) has been documented in laboratory settings,^14^ its occurrence and frequency in natural high-density infestations remain poorly characterised. Recent studies have highlighted how physiological trade-offs, such as those imposed by *T. cruzi* infection, can alter reproductive efficiency,^15^ suggesting that reproductive strategies in the wild may be plastic and responsive to environmental stress.

In this study, we employed exhaustive sampling and high-resolution microsatellite genotyping to deconstruct the genealogy of a massive *T. infestans* infestation in the Bolivian Chaco. We aimed to: (1) determine the number of discrete families within a domiciliary focus, (2) characterise the in-situ mating systems (monogamy vs polygamy), and (3) estimate the differential growth rates of these lineages to infer intra-colonial competition.

## MATERIALS AND METHODS

*Ethics* - The collection of triatomines was performed in accordance with the regulations of the Bolivian Ministry of Health. Informed consent was obtained from the head of each household prior to entering the dwellings for entomological inspection. No experiments on humans or animals were conducted in this study.

**Fig. 1:**
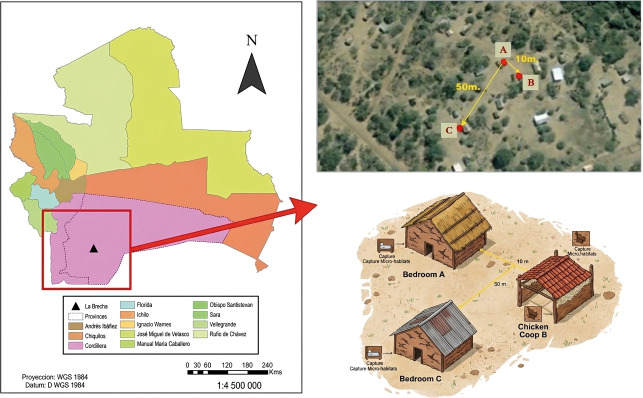
eco-epidemiological characterisation and spatial configuration of the study site in La Brecha. (A) Regional context showing the geographical location of the study area within the Cordillera province, Santa Cruz Department, Bolivia (Projection: WGS 1984). (B) Satellite detail providing a high-resolution view of the precise spatial distribution and relative distances (10 m and 50 m) between the sampled units A, B, and C. (C) Structural connectivity illustrated by an isometric diagram showing the link between domiciliary units (Bedrooms A and C) and the peridomestic environment (Chicken coop B). Insets highlight specific micro-habitats (beds, wall cracks, and nesting boxes) where *Triatoma infestans* aggregates were sampled. Yellow arrows denote the spatial proximity between ecotopes, representing the potential pathways for active insect dispersal.

*Study area and sampling* - Fieldwork was conducted in the community of La Brecha (19º30'S, 62º34'W), Santa Cruz, Bolivia. Three neighbouring structures were sampled: two domiciliary units (Bedrooms A and C) and one peridomiciliary structure (Chicken coop B). Insects were collected using the man-hour effort method (1 h per structure) by two experienced technicians. Active search was exhaustive and targeted specific micro-habitats known as refuges: intra-wall cracks, behind hanging objects (clothes, pictures), and within bed structures (mattresses and frames). A total of 381 specimens were collected and taxonomically identified as *T. infestans* following the keys of Lent and Wygodzinsky^2^ (Fig. 1).

*Microsatellite genotyping and data quality* - Genomic DNA was extracted from the legs of the insects using the cetyltrimethylammonium bromide (CTAB) 2% protocol. We amplified eight highly polymorphic microsatellite loci (TiA02, TiC02, TiC08, TiC09, TiD09, TiE02, TiE12, TiFO3) via polymerase chain reaction (PCR). These loci, specifically designed for *T. **infestans***,^16^ were previously validated for their high polymorphism information content (PIC) and substantial power of exclusion in Gran Chaco populations.^17^ PCR products were sized on an ABI PRISM 377 automated sequencer relative to a GeneScan-500 ROX internal standard [Supplementary data (Table)].

To ensure statistical robustness, we checked for genotyping errors, potential scoring inaccuracies, and the presence of null alleles using Micro-Checker.^18^ Genetic diversity parameters, including the probability of identity (PID) and exclusion probabilities, were calculated using GenAlEx v6.5.^19^ Global Hardy-Weinberg equilibrium and linkage disequilibrium were verified using GenePop v4.7,^20^ and the inbreeding coefficient (*F*_IS_) were calculated according to the methods of Weir and Cockerham.^21^ The loci panel showed high informative content (Mean *H*_E_ = 0.675), providing a combined exclusion probability > 0.999, which is sufficient to discriminate full-siblings from half-siblings with high confidence.

*Genealogical and demographic reconstruction* - Sibship and mating systems were reconstructed using a maximum likelihood (ML) approach in COLONY v2.0.^22^,^23^ We assumed a polygamous model for both sexes (polygyny and polyandry) without prior kinship information. Parameters included a full-likelihood method with medium run length, high likelihood precision, and a genotyping error rate of 0.01.

To estimate the demographic fitness of the inferred lineages, the finite growth rate (λ) was calculated for each family. This metric was derived from a stage-structured analysis where the transition probabilities between specific nymphal stages (*N*_t_ to *N*_t+1_) were computed to obtain the geometric mean of growth for each lineage. This approach allowed us to quantify the differential reproductive success between monogamous and polygamous families within the same environmental context.

## RESULTS

*Genetic diversity and marker resolution* - The eight microsatellite loci revealed substantial genetic variability, validating their resolving power for pedigree analysis. The average number of alleles per locus was 8.63 (± 3.58), with an expected heterozygosity (*H*_E_) of 0.675. Global Hardy-Weinberg equilibrium tests revealed a significant heterozygote deficiency, indicated by the inbreeding coefficient (*F*_IS_ = 0.0015, p < 0.05) at the colony level. This deviation is consistent with the Wahlund effect, confirming that the aggregate is not a single panmictic unit, but a subdivided population structured into distinct family clans.^21^

**Fig. 2:**
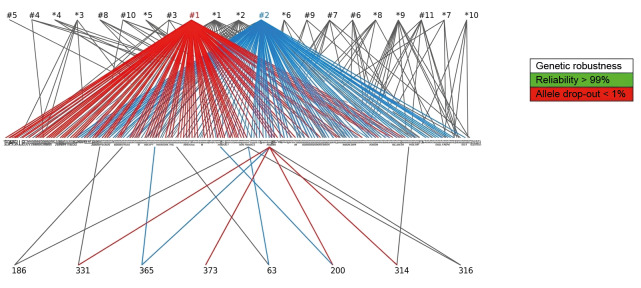
genealogical reconstruction of the *Triatoma infestans* colony. Pedigree inferred via maximum likelihood (ML) (COLONY v2.0) based on eight microsatellite loci. The colony is structured into 42 full-sibling families. Dominant families F1 (red) and F2 (blue) show expansive, multi-generational lineages, whereas minor families (grey) appear as fragmented, shallow clusters indicative of recent immigration or low reproductive success.

**Fig. 3:**
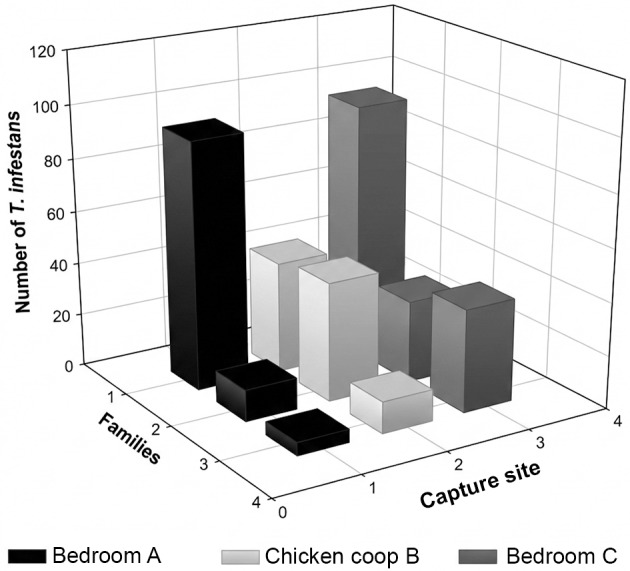
abundance distribution of *Triatoma infestans* families. Family identifiers (F1 to F42) are shown on the X-axis. A stark disparity in reproductive success is observed: families F1 and F2 (predominantly monogamous origin) account for the majority of the population (n > 80), while the remaining 40 families (polygamous origin) persist at low densities (n < 5).

*Genealogical architecture: the "mosaic" structure* - Pedigree reconstruction revealed that the infestation was organised into 42 distinct full-sibling families originating from 21 founder progenitors (11 females and 10 males). We identified a stark dichotomy in reproductive strategies (Table):

- Dominant lineages: two families (F1 and F2) comprised 84% of the population (n = 232 and n = 88, respectively). These lineages were characterised by a stable monogamous origin.

- Minor lineages: the remaining 40 families were low-abundance clusters (n = 2-4) characterised by high promiscuity, with polygyny and polyandry detected in 95% of these groups (Figs 2-3).

**TABLE:** Demographic and genetic characterisation of the reconstructed *Triatoma infestans* families in La Brecha, Bolivia

Family ID	Abundance (*N*)	Founders (*N*_m_, *N*_f_)	Mating system	Micro-habitat preference*	Genetic status (bottleneck test)	Finite growth rate (λ)
Family 1 (F1)	232	4, 3	Monogamy (stable)	Domicile (bedrooms) and peridomicile	Expansion (*H*_E_ < *H*_eq_)	1.03
Family 2 (F2)	88	2, 2	Monogamy (stable)	Domicile and peridomicile	Bottleneck (*H*_E_ > *H*_eq_)	1.22
Minor families (pooled)	61	4, 6	Polygyny/polyandry	Peridomicile (chicken coop)	Equilibrium/undefined	0.98 (avg)
Total colony	381	10, 11	Mixed	-	-	1.07

*Micro-habitat preference indicates where > 60% of family members were collected. Founders: number of inferred male (*N*_m_) and female (*N*_f_) parents. Genetic status inferred from heterozygosity excess/deficiency tests under TPM/SMM models. λ: finite rate of increase derived from stage-structured matrices.

*Divergent demographic histories (expansion vs bottleneck)* - Beyond abundance, the dominant families displayed contrasting genetic signatures indicative of different evolutionary histories. Family F1 exhibited a significant heterozygosity deficit (*H*_E_ < *H*_eq_) under the stepwise mutation model (SMM), a genetic signature typical of recent population expansion. In contrast, family F2 showed a significant heterozygosity excess (*H*_E_ > *H*_eq_) under the infinite allele model (IAM), which is indicative of a recent genetic bottleneck.^24^,^25^

**Fig. 4:**
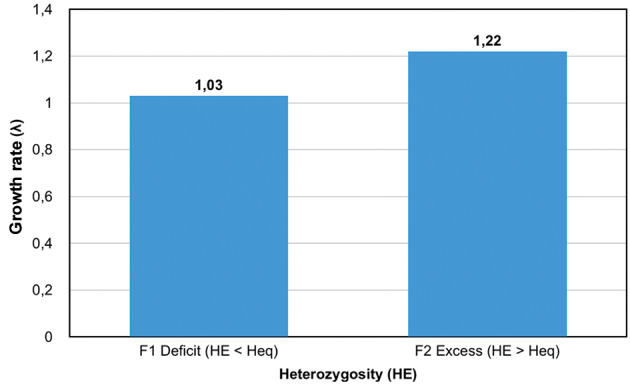
divergent evolutionary histories of the dominant lineages. (A) Genetic signatures: family 1 shows heterozygosity deficiency consistent with expansion, while family 2 shows excess consistent with a recent bottleneck. (B) Demographic fitness: family 2 exhibits a significantly higher finite growth rate (λ = 1.22) compared to family 1 (λ = 1.03), suggesting a "resilience" strategy where post-bottleneck lineages invest heavily in rapid population recovery.

*Demographic fitness and competition* - The demographic analysis confirmed active intra-colonial competition. While the overall colony showed a growth rate of λ = 1.07, the bottleneck-recovering family F2 exhibited a significantly higher finite growth rate (λ = 1.22) compared to the expanding family F1 (λ = 1.03). This suggests a process of ecological replacement, where F2 — despite being less abundant at the time of sampling — demonstrates superior reproductive performance (Fig. 4).

## DISCUSSION

The fine-scale genealogical reconstruction of *T. infestans* in La Brecha reveals a complex social mosaic that challenges the operational assumption of genetic uniformity in domiciliary infestations. Our findings reveal a cryptic social structure characterised by the coexistence of monogamous dominant lineages and a "tail" of numerous polygamous, low-abundance families.

*Mating systems and the founder effect paradox* - Reviewers questioned whether the dominance of families F1 and F2 represents an adaptive advantage or simply a founder effect. Our genetic data supports a more complex scenario. The bottleneck signature found in family F2 suggests it is a survivor lineage — what we term a 'resilient family' — that is now recovering (λ = 1.22). In contrast, family F1 shows signs of aggressive expansion. This coexistence of expansion and recovery strategies within the same domicile supports the hypothesis that reinfestation is driven by a mosaic of families with different demographic histories, rather than a simple random accumulation of individuals.

The presence of multiple mating strategies within a single colony presents a 'paradox of polygamy' in the domestic environment. While polyandry in insects is often a strategy to increase genetic diversity and offspring fitness,^26^ our results show that the most numerically successful families (F1 and F2) were derived from stable monogamous events. The mating behaviour and stridulation patterns in *T. infestans*, which facilitate these encounters, have been well-documented.^14^ However, as proposed by Lobbia et al.,^15^ biological costs and physiological trade-offs can alter these reproductive choices under specific environmental pressures. This suggests that in high-density domestic colonies, monogamy might provide a competitive advantage by stabilising lineage expansion.

*Resolving power and marker sensitivity* - Regarding the resolution of our markers, the high polymorphism of the eight microsatellite loci provided sufficient power to discriminate first-order relatives within these aggregates. Previous studies using different markers suggested that the panmictic unit of *T. infestans* is significantly smaller than previously assumed;^27^,^28^,^29^,^30^ our genealogical data confirm this micro-structural subdivision. The detection of polygyny and polyandry in 40 out of 42 families demonstrates that our marker set was sensitive enough to capture complex reproductive events, even on a small spatial scale.

*Implications for vector control and "One health"* - The existence of multiple competing families has profound implications for public health. A focal insecticide spray might eliminate conspicuous, dominant families but miss cryptic; minor lineages hidden in deep crevices or peridomestic structures.^31^ These survivors could then experience a 'competitive release,' leading to rapid reinfestation.^32^ Furthermore, active dispersal by walking, particularly by females, has been identified as a critical factor in house reinfestation.^33^,^34^ The genetic connectivity found between the chicken coop and the bedrooms confirms that peridomestic structures act as permanent reservoirs that jeopardize control efforts.^35^ This supports the need for 'One health' approaches that manage the entire domestic-peridomestic interface as a single unit.

*In conclusion* - This study provides the first fine-scale genealogical deconstruction of a massive *T. infestans* infestation. We conclude that:

*Social complexity* - Domiciliary colonies are structured as a "mosaic of families" where monogamy and polygamy coexist as plastic reproductive strategies. Monogamy appears associated with dominant, established lineages, while promiscuity characterises minor, potentially colonising families.

*Demographic resilience* - The genetic evidence of simultaneous population expansion (F1) and recovery from bottlenecks (F2) within the same household confirms that reinfestation is not a uniform process but involves lineages with different histories and competitive abilities.

*Implications for control* - The existence of "cryptic" minor families and the high connectivity between peridomestic and domestic structures challenges the efficacy of focal spraying. Control interventions must be broad-spectrum and spatially inclusive ("One health" approach) to eliminate both the dominant/visible families and the resilient/minor lineages that drive recurrence.

## SUPPLEMENTARY MATERIALS

Supplementary data

## Data Availability

The contents underlying the research text are included in the manuscript.
