# Supplementary material for: Coexistence of monogamy and polygyny in Triatoma infestans: fine-scale genealogical structure reveals complex social structures within domestic colonies in the Bolivian Chaco
Source: Mem Inst Oswaldo Cruz. 2026 Jun 15;121:e250355. doi: 10.1590/0074-02760250355 (PMC13268354; doi:10.1590/0074-02760250355)
Supplement: Supplementary data [file 1678-8060-mioc-121-e250355-s1.pdf]

TABLE

Genotypic data of *Triatoma infestans* individuals from domestic and peridomestic ecotopes in the Bolivian Chaco, based on eight microsatellite loci

			Microsatellite loci															
ID	Stage	Structure	A02_1	A02_2	E12_1	E12_2	F03_1	F03_2	C08_1	C08_2	E02_1	E02_2	C02_1	C02_2	D09_1	D09_2	C09_1	C09_2
1	N1	A	192	192	300	312	172	204	205	205	157	161	183	183	209	209	133	135
2	N1	A	170	198	300	312	178	204	205	205	157	161	177	183	205	209	133	139
3	N1	A	170	182	300	314	172	202	203	205	149	161	177	183	205	209	133	139
4	N1	A	170	198	300	300	172	204	203	205	157	161	177	183	197	209	133	139
5	N1	A	170	198	300	300	172	178	205	205	149	161	177	183	197	205	139	139
6	N1	A	170	222	300	312	178	182	205	205	149	149	177	177	197	197	139	139
7	N2	A	170	222	300	312	172	202	203	205	149	149	173	177	197	217	139	159
8	N2	A	198	198	0	0	172	178	205	205	149	161	177	177	197	209	139	139
9	N2	A	170	198	308	308	172	178	205	205	149	161	177	183	197	209	139	139
10	N2	A	184	198	300	312	178	182	205	205	149	149	173	183	209	233	139	159
11	N2	A	170	198	300	312	172	204	205	205	149	157	183	183	209	233	133	139
12	N2	A	182	182	300	312	172	204	205	205	149	161	169	183	209	209	131	139
13	N2	A	198	198	300	312	172	178	205	205	149	161	173	177	209	241	139	139
14	N3	A	170	198	300	300	172	178	203	205	149	157	175	175	197	217	139	139
15	N3	A	170	224	300	312	172	182	203	205	149	149	175	183	197	205	139	139
16	N3	A	170	196	300	312	172	204	199	205	149	161	177	177	209	233	131	131
17	N3	A	0	0	300	312	172	204	205	205	149	157	177	199	197	209	139	139
18	N3	A	192	192	300	312	172	178	205	205	149	161	177	177	209	209	131	157
19	N3	A	170	198	312	312	172	178	203	205	149	149	169	173	209	209	139	157
20	N3	A	198	198	300	312	172	182	203	205	149	161	177	199	209	209	139	139
21	N3	A	170	224	300	312	172	204	205	205	149	157	177	183	197	209	139	139
22	N4	A	170	224	300	312	172	204	203	205	149	149	173	177	209	241	139	139
23	N4	A	192	192	300	312	178	204	205	205	157	161	169	177	209	209	139	139
24	N5	A	170	224	300	312	172	204	205	205	149	161	177	199	205	209	139	139
25	N1	A	182	182	300	312	172	172	203	205	149	161	169	183	197	209	157	159
26	N2	A	170	224	300	312	172	204	205	205	149	161	183	183	197	197	133	139
27	N2	A	198	198	300	300	172	178	205	205	149	161	169	183	197	213	139	157
28	N2	A	170	222	300	312	172	204	205	205	157	161	173	177	217	233	139	139
29	N2	A	0	0	300	312	172	202	203	203	149	161	177	199	209	233	133	139
30	N3	A	170	224	300	312	172	204	203	205	149	161	177	183	209	217	139	139
31	N4	A	170	224	300	312	172	204	203	205	149	149	177	183	209	209	139	139
32	M	A	182	186	300	314	172	178	205	205	149	161	177	177	197	209	133	159
33	N1	A	170	224	312	312	204	204	203	205	149	157	177	183	209	209	139	139
34	N1	A	170	198	300	314	204	204	205	205	149	161	177	177	209	233	133	139
35	N2	A	170	224	300	308	204	204	205	205	149	161	173	183	197	241	139	139
36	N3	A	170	198	300	312	204	204	205	205	149	149	173	183	197	209	139	139
37	N3	A	170	198	300	312	172	182	203	205	149	149	161	183	209	209	139	139
38	N3	A	196	196	300	312	172	204	205	205	149	161	175	183	209	233	131	159
39	N4	A	170	192	300	300	172	178	203	205	149	149	173	183	197	205	139	139
40	M	A	170	224	300	300	172	178	203	205	149	161	177	199	197	209	131	139
41	N1	A	192	198	300	300	172	204	205	205	157	161	183	183	197	209	131	135
42	N1	A	170	170	300	312	172	172	205	205	149	149	177	183	197	233	159	159
43	N2	A	170	198	300	312	172	178	205	205	149	161	173	175	209	217	139	139
44	N2	A	170	198	300	312	172	178	205	205	149	157	175	183	209	209	131	139
45	N2	A	198	198	312	312	172	182	203	205	149	157	183	183	217	233	139	159

			Microsatellite loci															
ID	Stage	Structure	A02_1	A02_2	E12_1	E12_2	F03_1	F03_2	C08_1	C08_2	E02_1	E02_2	C02_1	C02_2	D09_1	D09_2	C09_1	C09_2
46	N2	A	0	0	300	314	172	204	205	205	157	161	183	183	197	209	131	135
47	N2	A	198	198	300	312	178	182	205	205	149	157	173	183	213	233	139	159
48	N2	A	170	198	300	312	172	204	205	205	149	161	183	183	197	205	131	139
49	N2	A	170	222	300	312	172	204	205	205	149	149	173	183	209	209	133	159
50	N2	A	198	198	300	300	172	204	205	205	149	149	169	183	197	209	139	159
51	N2	A	198	198	300	312	172	204	205	205	149	149	177	183	197	233	139	139
52	N2	A	198	198	300	312	172	178	205	205	149	149	173	183	197	209	139	139
53	N2	A	198	198	300	300	178	204	205	205	149	149	183	183	209	209	139	159
54	N2	A	198	198	300	312	172	204	205	205	157	161	177	177	209	217	131	139
55	N2	A	170	198	300	312	172	204	203	205	157	161	177	183	197	209	131	139
56	N2	A	170	222	300	300	172	204	205	205	149	161	177	183	209	233	131	159
57	N2	A	170	198	300	300	172	178	205	205	149	161	183	183	209	209	131	139
58	N2	A	170	222	312	312	172	178	205	205	149	157	183	183	217	233	139	159
59	N2	A	170	170	300	312	172	182	205	205	149	161	183	183	197	217	131	159
60	N2	A	170	224	300	312	172	204	205	205	149	161	177	177	197	197	131	139
61	N2	A	170	224	300	312	172	172	203	205	149	157	183	199	233	241	139	159
62	N2	A	170	198	312	312	172	182	205	205	149	149	183	183	197	197	139	159
63	N2	A	196	196	300	300	172	204	205	205	149	161	177	199	197	241	131	139
64	N2	A	198	198	300	312	172	178	205	205	149	157	183	183	197	233	139	139
65	N3	A	170	192	300	312	172	182	205	205	149	149	177	183	209	241	159	159
66	N3	A	182	198	300	300	172	178	199	205	149	161	177	199	197	233	131	159
67	N3	A	192	198	300	312	172	178	205	205	157	161	183	183	209	209	139	139
68	N3	A	170	170	300	300	172	204	205	205	149	161	183	183	209	233	159	159
69	N3	A	198	198	312	314	172	204	205	205	149	157	177	177	197	233	139	159
70	N3	A	170	198	300	312	172	182	203	205	157	161	173	199	205	209	131	139
71	N4	A	0	0	300	312	172	204	205	205	157	161	177	177	209	233	131	159
72	N4	A	198	198	300	312	172	178	205	205	157	157	177	183	217	233	139	139
73	M	A	170	222	300	312	172	182	203	205	149	149	183	199	209	209	139	139
74	M	A	192	192	300	312	178	204	205	205	149	157	183	199	197	205	139	159
76	N5	A	170	192	300	300	172	182	203	205	149	161	183	183	197	197	139	139
77	N5	A	170	170	300	314	172	172	205	205	149	161	177	177	197	209	139	139
78	H	A	170	222	300	300	172	204	205	205	157	161	177	177	197	213	131	139
79	H	A	198	198	300	312	178	182	205	205	149	161	173	199	209	241	139	139
80	H	A	192	192	300	312	172	178	205	205	149	161	163	173	209	209	139	139
81	M	A	198	198	300	312	178	204	203	205	149	161	177	177	209	233	131	139
82	M	A	0	0	300	312	172	178	205	205	157	161	183	199	209	241	139	139
83	N4	A	170	192	312	312	172	204	205	205	157	161	177	183	209	209	131	131
84	N5	A	170	222	300	312	172	178	203	205	157	161	177	183	197	209	139	139
85	N5	A	170	222	300	312	172	178	205	205	149	157	173	173	209	217	139	139
86	N5	A	198	198	300	312	172	178	205	205	149	157	177	183	217	233	139	139
87	N5	A	170	222	300	300	172	204	205	205	149	157	177	183	209	209	139	139
88	N5	A	192	198	300	312	172	178	205	205	149	161	177	177	197	209	139	159
89	N5	A	170	198	300	312	172	204	205	205	149	161	177	199	197	209	139	139
90	H	A	198	198	300	312	178	204	205	205	157	161	173	183	197	209	139	139
91	M	A	170	198	300	312	172	182	205	205	157	161	183	199	209	209	131	139
92	M	A	192	192	300	312	172	178	205	205	149	149	163	183	209	233	139	159
93	N3	A	170	222	0	0	172	182	203	205	157	161	173	199	205	209	139	139
94	N4	A	0	0	312	312	172	182	205	205	149	149	177	177	217	241	139	159

ID	Stage	Structure	Microsatellite loci															
			A02_1	A02_2	E12_1	E12_2	F03_1	F03_2	C08_1	C08_2	E02_1	E02_2	C02_1	C02_2	D09_1	D09_2	C09_1	C09_2
95	N4	A	198	198	300	312	172	204	205	205	149	149	177	177	233	233	139	159
96	N5	A	170	224	300	312	172	182	203	205	149	149	183	183	197	209	139	159
97	N5	A	170	222	300	312	178	202	203	205	149	149	173	183	209	241	139	139
98	N5	A	198	198	306	308	172	204	205	205	149	157	183	199	197	209	139	139
99	H	A	198	198	300	312	172	178	205	205	149	161	183	183	209	217	131	139
100	M	A	170	198	300	312	178	182	203	205	149	161	177	199	209	209	139	139
101	M	A	170	222	300	312	172	178	205	205	157	161	173	175	205	217	139	159
102	N4	A	170	198	300	312	172	182	205	205	149	161	183	199	233	241	139	159
103	H	A	170	192	300	312	172	182	205	205	157	161	173	175	209	209	131	139
104	H	A	170	192	300	312	178	204	205	205	149	157	169	169	197	209	139	139
105	N3	A	186	196	312	312	178	182	205	205	149	149	177	199	209	233	139	139
106	N5	A	170	222	300	312	172	178	203	205	149	161	183	199	205	209	139	139
107	N5	A	198	198	312	312	172	204	205	205	149	161	175	183	197	213	139	139
108	N5	A	192	198	300	312	172	182	205	205	149	161	177	183	209	213	131	139
109	N5	A	198	198	300	312	178	204	203	205	149	161	173	177	209	209	131	139
110	N5	A	170	198	300	312	172	182	205	205	149	161	173	199	197	209	139	139
111	N5	A	170	222	300	312	172	182	205	205	157	161	177	199	197	209	133	139
112	N5	A	190	190	300	312	172	178	205	205	149	149	177	183	197	209	139	139
113	H	B	198	198	308	312	178	182	203	205	149	161	177	183	197	213	139	139
114	H	B	170	222	300	312	172	172	203	205	149	161	173	183	197	217	139	139
115	H	B	168	196	300	312	178	204	205	205	153	153	173	177	209	209	139	161
116	H	B	190	198	300	312	172	178	205	205	157	157	177	183	209	209	133	135
117	H	B	170	170	300	312	172	172	205	205	149	157	173	183	197	217	135	139
118	H	B	170	190	308	312	172	182	205	205	149	149	177	183	209	241	139	139
119	M	B	170	198	300	312	172	178	205	205	157	161	177	177	209	217	139	139
120	M	B	170	170	300	312	178	204	205	205	157	161	173	183	209	217	139	139
121	M	B	170	198	300	312	172	204	205	205	149	157	173	173	209	233	133	139
122	N5	B	170	198	300	312	172	178	203	205	157	161	173	177	209	209	139	139
123	N5	B	198	198	300	312	172	172	203	205	149	161	183	183	197	209	135	135
124	N5	B	170	198	300	312	172	204	205	205	149	161	173	199	213	217	133	139
125	N5	B	198	198	300	312	172	182	203	203	157	157	173	177	209	213	133	139
126	N5	B	198	198	300	312	172	182	203	205	157	161	177	177	197	217	139	139
127	N5	B	170	222	300	300	172	204	205	205	149	157	173	173	197	209	139	139
128	N5	B	170	198	300	312	172	204	205	205	149	161	177	183	209	213	135	139
129	H	B	170	222	300	312	178	204	205	205	149	149	173	173	217	217	139	139
130	H	B	170	198	300	312	172	182	205	205	149	157	177	183	209	233	133	149
131	H	B	170	222	300	312	172	204	205	205	157	161	173	177	209	209	133	139
132	M	B	198	198	300	312	172	178	205	205	149	161	173	183	197	205	133	139
133	M	B	198	198	300	312	172	178	203	205	157	161	183	183	197	209	135	139
134	M	B	170	222	300	308	172	178	205	205	157	161	173	173	209	209	133	139
135	M	B	198	198	300	312	172	178	205	205	157	161	173	177	209	209	135	139
136	N5	B	170	222	312	312	182	186	203	205	157	161	177	199	205	209	139	139
137	N5	B	170	222	312	312	172	182	205	205	157	161	177	177	209	233	139	139
138	N5	B	170	222	300	312	172	178	205	205	157	161	173	173	197	209	135	139
139	H	B	198	198	300	312	172	172	203	203	157	157	173	173	205	209	135	139
140	H	B	192	192	300	300	178	204	203	205	149	149	183	197	197	239	139	139
141	M	B	192	198	300	312	172	182	205	205	149	157	175	183	209	233	139	139
142	M	B	198	198	300	312	172	172	203	205	157	157	177	177	209	217	135	139

			Microsatellite loci															
ID	Stage	Structure	A02_1	A02_2	E12_1	E12_2	F03_1	F03_2	C08_1	C08_2	E02_1	E02_2	C02_1	C02_2	D09_1	D09_2	C09_1	C09_2
143	M	B	0	0	300	312	172	204	205	205	157	161	173	175	209	209	139	139
144	N4	B	192	198	300	300	172	204	205	205	149	157	177	177	209	217	139	149
145	N5	B	170	222	300	312	178	204	205	205	157	161	173	183	209	209	139	139
146	N5	B	198	198	300	300	172	182	203	205	149	149	173	173	209	233	133	133
147	N5	B	0	0	300	300	178	204	205	205	149	157	175	183	197	209	139	139
149	N5	B	170	222	300	312	172	182	205	205	157	161	183	183	197	209	139	139
150	N5	B	170	222	300	312	178	182	205	205	157	161	173	197	209	209	139	139
151	N5	B	170	222	300	314	172	178	205	205	157	161	177	177	209	209	139	139
152	N5	B	170	198	300	312	172	204	203	205	157	157	173	177	209	213	139	139
153	N5	B	198	198	300	312	172	182	205	205	149	157	183	199	209	209	139	139
154	H	B	198	198	300	312	178	182	203	203	149	161	173	183	209	217	139	139
155	H	B	192	198	300	312	172	204	205	205	149	161	177	199	209	209	139	139
156	H	B	192	192	300	312	182	204	205	205	149	157	183	183	209	209	139	161
157	H	B	198	198	300	300	172	182	203	203	157	157	173	173	209	233	139	139
158	H	B	198	198	300	312	172	178	203	205	149	149	183	183	209	233	139	161
159	H	B	198	198	300	312	172	204	203	205	157	161	173	177	197	209	133	139
160	H	B	192	192	300	312	178	204	205	205	149	161	177	183	197	209	139	139
161	H	B	170	222	300	312	178	182	203	205	149	161	177	183	209	213	139	139
162	H	B	170	198	300	312	172	178	203	205	149	149	177	177	209	217	139	139
163	M	B	198	198	300	312	172	204	203	205	157	161	177	177	209	217	139	139
164	M	B	170	198	300	312	172	182	205	205	157	161	183	199	213	217	135	135
165	M	B	170	222	312	312	172	204	205	205	149	149	173	183	197	217	139	139
166	M	B	192	198	300	312	172	204	203	205	157	161	183	199	209	209	133	139
167	M	B	170	222	300	312	172	178	205	205	149	161	173	177	197	209	139	139
168	M	B	170	198	300	312	178	182	205	205	157	161	173	177	205	209	139	139
169	N4	B	170	222	300	312	172	178	203	205	157	161	183	205	201	217	135	135
170	N5	B	192	198	300	312	172	178	205	205	149	149	185	185	197	197	139	161
171	N5	B	170	198	300	300	172	172	203	205	157	161	177	177	197	233	135	135
172	N5	B	170	198	300	312	172	182	205	205	157	161	177	185	209	213	135	135
173	N5	B	198	198	300	312	172	172	203	205	157	157	175	177	197	213	139	139
174	N5	B	0	0	300	312	172	178	203	205	149	149	177	199	209	233	139	139
175	N5	B	170	222	300	312	178	182	205	205	149	161	177	177	209	217	139	139
176	N5	B	198	198	300	312	172	202	203	203	149	157	179	201	209	213	139	139
177	N5	B	170	198	300	308	172	204	205	205	149	157	179	185	213	217	139	139
178	N5	B	192	192	300	312	172	178	205	205	149	149	173	177	209	209	139	139
179	N5	B	198	198	312	314	172	178	203	205	149	157	177	185	197	209	139	161
180	N5	B	198	198	300	312	172	178	203	205	149	161	177	177	197	213	133	133
181	H	B	0	0	300	308	172	178	203	205	149	161	175	199	197	213	133	139
182	H	B	198	198	300	312	178	202	203	203	157	161	173	175	197	209	133	139
183	H	B	170	222	300	312	172	204	203	205	149	161	177	183	217	233	139	139
184	H	B	196	196	300	300	172	204	203	205	149	157	179	179	197	221	139	161
185	H	B	186	196	300	308	178	182	205	205	149	161	173	177	209	217	139	139
186	M	B	198	198	312	314	172	182	205	205	149	149	179	179	217	241	139	149
187	M	B	192	198	300	312	172	204	205	205	157	157	163	173	205	209	139	139
188	M	B	192	198	300	312	172	182	203	205	149	157	177	177	197	197	139	139
189	M	B	170	192	300	312	172	178	205	205	149	161	177	199	197	197	133	139
190	M	B	170	224	300	312	172	182	205	205	149	149	177	199	197	209	139	139
191	M	B	170	224	300	312	172	204	205	205	149	161	175	177	209	241	133	139

			Microsatellite loci															
ID	Stage	Structure	A02_1	A02_2	E12_1	E12_2	F03_1	F03_2	C08_1	C08_2	E02_1	E02_2	C02_1	C02_2	D09_1	D09_2	C09_1	C09_2
192	M	B	170	192	300	312	172	204	203	205	149	157	179	179	199	199	139	161
193	M	B	170	198	312	312	172	178	203	203	149	149	183	205	201	217	139	159
194	M	B	170	224	300	312	172	182	201	203	161	161	177	199	197	209	131	139
195	N4	B	184	184	300	312	172	204	203	203	149	161	177	183	209	213	131	149
196	N5	B	192	198	300	312	178	182	203	203	149	157	177	177	209	209	139	139
197	N5	B	184	184	300	312	172	182	201	203	161	161	177	183	209	209	131	131
198	N5	B	192	192	300	312	178	182	201	203	149	161	175	197	209	209	131	139
199	N5	B	170	224	300	312	182	204	201	203	149	149	175	175	197	209	139	159
200	N5	B	170	198	300	312	172	182	201	203	161	161	175	177	209	209	139	139
201	N5	B	170	224	312	312	172	178	201	203	157	161	177	177	209	233	139	139
202	N5	B	170	198	300	312	178	204	203	203	149	161	177	177	209	209	147	159
203	N5	B	192	192	300	312	178	182	201	203	149	161	175	197	209	209	131	139
204	N5	B	198	198	308	312	172	178	203	203	149	149	181	181	209	217	139	139
205	N5	B	190	190	312	314	172	182	201	203	157	161	0	0	205	209	139	139
206	N5	B	170	198	300	312	178	182	203	203	149	161	177	177	197	197	139	139
207	N5	B	170	198	300	312	178	202	201	201	149	149	177	177	197	217	131	159
208	H	B	192	198	300	300	172	178	203	203	157	161	165	185	213	221	131	139
209	H	B	170	198	300	312	172	204	203	203	157	157	179	179	205	209	139	139
210	M	B	190	190	300	312	172	178	203	203	149	157	177	177	209	217	139	147
211	N5	B	170	224	300	308	172	204	203	203	157	161	173	183	209	217	131	139
212	N5	B	170	192	300	312	172	204	201	203	149	157	177	177	197	197	139	159
213	N1	B	170	198	300	312	172	182	201	203	149	149	173	197	217	233	139	139
214	N1	C	0	0	300	312	178	202	201	201	149	157	177	183	197	217	139	159
215	N1	C	170	224	300	312	172	178	203	203	157	157	177	177	209	217	139	139
216	N1	C	170	192	300	312	178	182	203	203	157	161	183	183	209	209	131	135
217	N1	C	192	198	300	312	178	204	203	203	149	149	177	177	209	217	131	139
218	N1	C	170	192	300	312	172	178	201	203	157	161	183	183	209	233	139	139
219	N1	C	170	224	300	308	172	178	203	203	149	161	183	199	209	209	139	147
220	N2	C	170	224	300	308	178	202	201	201	149	149	177	183	197	209	159	159
221	N2	C	192	192	300	312	178	182	201	203	149	157	175	177	209	217	139	139
222	N2	C	170	224	312	314	178	182	203	203	149	157	173	177	209	209	139	139
223	N2	C	170	224	300	312	172	182	201	203	157	161	177	177	205	209	131	139
224	N2	C	198	198	300	312	178	182	201	203	149	149	177	199	197	217	139	159
225	N2	C	170	198	300	312	178	204	203	203	149	161	177	199	197	233	139	139
226	N2	C	170	224	300	312	178	182	201	201	149	149	183	183	233	241	139	139
227	N2	C	170	224	300	312	172	178	201	203	157	161	183	197	197	213	131	139
228	N2	C	170	224	300	312	172	172	201	203	149	161	173	199	217	233	139	139
229	N2	C	192	198	312	314	178	182	203	203	149	149	173	177	209	209	139	159
230	N2	C	198	198	300	312	172	178	201	203	157	161	173	177	209	217	139	159
231	N2	C	184	192	312	312	172	178	203	203	149	157	183	183	197	217	139	139
232	N3	C	170	192	300	312	172	178	203	203	149	149	177	183	209	213	139	159
233	N2	C	192	192	300	312	172	178	201	203	157	157	177	177	209	209	139	147
234	H	C	192	192	300	300	178	182	201	203	157	161	177	177	205	233	131	139
235	H	C	192	192	300	314	178	182	201	203	149	161	183	199	197	213	131	159
236	M	C	170	222	312	312	172	178	201	203	157	161	173	199	209	209	131	131
237	M	C	170	192	300	312	172	204	203	203	157	161	177	183	205	209	135	139
238	M	C	192	192	300	300	178	182	201	203	149	149	185	199	197	209	139	139
239	N1	C	192	192	300	312	182	202	201	203	157	161	177	177	209	209	131	139

			Microsatellite loci															
ID	Stage	Structure	A02_1	A02_2	E12_1	E12_2	F03_1	F03_2	C08_1	C08_2	E02_1	E02_2	C02_1	C02_2	D09_1	D09_2	C09_1	C09_2
240	N3	C	192	198	300	312	172	204	203	203	157	157	177	177	197	209	139	147
241	N3	C	224	224	300	312	178	204	201	203	157	157	173	177	197	233	139	147
242	N3	C	170	192	312	312	172	182	201	203	149	157	177	183	197	205	139	147
243	N5	C	170	224	300	312	172	172	201	203	149	149	177	183	197	213	139	139
244	M	C	192	192	312	314	178	204	203	203	149	161	163	183	197	209	131	139
245	M	C	170	198	300	312	172	178	201	201	149	157	177	199	197	197	147	159
246	N1	C	192	192	300	308	172	204	203	203	157	161	173	199	209	213	139	139
247	N1	C	190	190	300	312	172	178	201	203	149	161	173	173	205	205	139	139
248	N1	C	170	224	300	314	172	178	203	203	149	149	177	183	205	205	0	0
249	N1	C	198	198	300	300	178	204	203	203	157	161	183	199	209	233	139	139
250	N1	C	170	224	300	300	172	172	201	203	149	157	177	183	197	209	139	139
251	N1	C	198	198	300	312	172	204	203	203	149	161	183	183	217	217	131	139
252	N1	C	170	198	300	312	172	204	203	203	149	161	177	183	233	241	131	139
253	N1	C	196	196	300	312	172	204	201	203	149	161	173	173	205	205	139	139
254	N1	C	192	198	300	312	172	204	201	203	149	165	177	199	209	209	131	159
255	N1	C	192	192	300	312	172	182	203	203	149	149	177	199	209	233	139	139
256	N1	C	170	192	300	312	172	178	203	203	157	161	173	177	209	209	131	139
257	N1	C	170	198	300	312	178	178	203	203	149	161	177	185	209	233	131	139
259	N1	C	170	224	300	300	172	204	203	203	157	161	177	183	197	217	131	139
260	N1	C	186	186	300	314	172	178	203	203	149	149	183	199	205	209	159	159
261	N1	C	170	192	300	312	178	182	201	203	149	165	177	183	209	233	131	139
262	N1	C	0	0	300	312	172	178	203	203	149	161	173	183	209	233	135	139
263	N1	C	198	198	300	312	172	204	203	203	149	157	177	199	197	205	139	139
264	N1	C	0	0	300	312	178	182	201	203	0	0	183	199	209	209	131	139
265	N1	C	170	224	300	312	172	178	201	203	149	149	183	199	209	209	139	139
266	N1	C	170	198	300	312	172	178	203	203	149	157	173	177	197	217	131	139
267	N1	C	170	192	300	312	172	178	201	203	149	149	183	199	197	205	139	159
268	N1	C	192	192	300	312	172	182	201	203	157	157	177	177	197	233	139	139
269	N1	C	192	192	300	312	172	178	201	203	149	149	177	199	197	209	139	159
270	N1	C	170	224	300	312	172	182	201	203	149	157	173	177	205	241	139	139
271	N1	C	170	224	300	312	172	182	203	203	149	161	173	177	213	217	131	139
272	N1	C	198	198	300	312	176	182	203	203	149	161	177	183	197	217	131	139
273	N1	C	192	198	300	314	172	178	203	203	149	157	163	183	197	209	139	139
274	N1	C	170	222	300	312	172	176	201	203	161	161	177	183	205	209	135	139
275	N1	C	170	192	312	312	176	202	203	203	149	157	177	183	197	217	139	147
276	N2	C	170	192	312	314	172	182	203	203	157	161	183	183	209	213	139	139
277	N2	C	192	192	308	312	172	182	0	0	149	149	0	0	209	209	0	0
278	N2	C	170	198	300	312	172	202	201	203	149	161	177	177	213	241	139	139
279	N2	C	184	192	300	312	172	176	201	203	157	161	173	177	205	233	131	139
280	N2	C	170	224	308	314	172	176	203	203	157	161	173	183	209	209	135	139
281	N2	C	170	224	300	312	172	182	201	203	157	161	177	177	209	233	131	139
282	N2	C	0	0	300	312	178	180	201	203	149	149	185	199	209	217	139	159
283	N2	C	170	224	300	312	172	176	201	203	149	161	173	199	205	209	135	139
284	N2	C	192	192	300	312	176	182	201	203	157	157	173	177	209	233	139	139
285	N2	C	198	198	300	312	172	202	203	203	149	161	177	183	197	217	131	139
286	N2	C	170	224	300	312	172	182	203	203	161	161	183	199	209	209	131	131
287	N2	C	0	0	300	314	172	176	203	203	157	161	197	199	217	233	139	159
288	N2	C	192	192	300	312	176	180	201	203	149	157	163	183	197	233	139	159

ID	Stage	Structure	A02_1	A02_2	E12_1	E12_2	F03_1	F03_2	C08_1	C08_2	E02_1	E02_2	C02_1	C02_2	D09_1	D09_2	C09_1	C09_2
			Microsatellite loci															
289	N2	C	170	192	312	312	172	176	201	203	149	149	173	183	197	209	139	139
290	N2	C	170	198	300	312	172	176	201	203	149	149	177	183	209	241	139	139
291	N2	C	198	198	300	300	172	180	203	203	157	161	177	185	213	233	139	139
292	N3	C	170	222	300	312	172	180	201	203	157	157	183	199	197	217	139	147
293	N3	C	192	192	300	312	172	178	203	203	149	149	177	177	197	209	139	159
294	N3	C	170	192	300	312	176	182	203	203	157	161	163	177	209	233	131	139
295	N3	C	192	192	312	312	172	172	202	203	157	161	173	183	217	217	131	139
296	N3	C	170	182	300	312	172	176	203	203	149	161	177	199	209	217	139	139
297	N3	C	170	198	312	312	172	176	201	203	157	161	163	183	197	209	131	131
298	N3	C	198	198	300	312	172	202	203	203	149	157	173	177	197	209	139	159
299	N3	C	170	192	300	312	176	182	201	203	161	161	177	183	209	233	131	139
300	N3	C	170	198	300	312	172	176	201	203	149	161	173	183	209	241	131	139
301	N3	C	184	192	300	312	182	204	203	203	161	165	173	177	201	209	131	139
302	N3	C	190	190	300	314	176	180	203	203	149	157	177	183	197	209	131	159
303	N3	C	170	222	300	312	172	176	203	203	149	161	173	177	209	209	139	139
304	N3	C	170	182	300	312	172	180	197	203	149	157	177	199	197	233	131	139
305	N3	C	170	198	300	300	172	176	201	201	149	161	177	183	197	209	131	159
306	N3	C	170	222	300	312	172	182	201	203	149	157	177	177	209	233	139	159
307	N3	C	192	192	312	312	176	176	0	0	149	157	183	199	205	209	139	139
308	N3	C	170	222	300	312	172	180	201	203	149	149	177	183	209	233	139	139
309	N3	C	170	192	300	300	172	176	201	203	157	161	177	199	209	209	131	139
310	N3	C	192	192	312	312	172	182	201	203	149	157	177	183	209	233	139	139
311	N3	C	170	190	312	312	172	202	203	203	149	157	177	199	209	233	139	139
312	N3	C	170	222	300	308	172	204	201	203	149	161	177	199	205	213	131	131
313	N4	C	196	196	300	314	172	178	201	203	149	161	177	177	197	213	139	139
314	N4	C	0	0	300	312	176	180	203	203	149	149	177	183	205	209	139	159
315	N4	C	170	198	300	312	172	178	201	203	149	157	173	183	197	233	139	139
316	N4	C	198	198	300	312	176	176	203	203	149	161	177	199	209	241	139	139
317	N5	C	192	192	300	312	172	202	203	203	149	157	177	183	209	209	139	139
318	N5	C	170	198	300	312	172	182	203	203	149	161	177	199	209	217	139	139
319	N5	C	170	198	300	312	172	202	203	203	149	161	173	177	217	217	139	139
321	M	C	170	222	300	312	172	202	203	203	157	161	173	177	209	217	131	139
322	M	C	198	198	300	312	176	182	203	203	149	161	177	177	209	209	139	139
323	M	C	170	198	300	312	172	180	203	203	149	161	177	177	0	0	135	159
324	M	C	190	198	300	312	172	180	201	203	149	157	173	177	197	209	139	139
325	N1	C	170	222	300	300	172	182	203	203	0	0	173	177	209	213	139	139
326	N1	C	198	198	300	312	172	182	203	203	157	161	173	173	209	233	139	139
327	N1	C	184	184	300	312	176	182	201	203	157	161	169	177	197	209	131	155
328	N1	C	170	198	300	312	172	182	203	203	149	161	177	177	209	233	135	139
329	N1	C	198	198	300	312	172	182	203	203	157	161	177	177	197	197	139	139
330	N1	C	170	198	300	314	172	180	203	203	157	161	173	177	217	217	135	135
331	N1	C	170	198	300	312	176	180	201	203	149	149	177	183	209	233	139	139
332	N1	C	0	0	312	312	182	202	203	203	161	161	177	177	213	213	135	135
333	N1	C	192	192	300	300	172	176	201	203	149	161	183	199	209	209	139	139
334	N1	C	198	198	300	312	172	176	203	203	149	149	173	183	197	209	139	159
335	N1	C	170	198	300	300	178	202	203	203	161	161	177	183	197	205	131	131
336	N1	C	170	222	300	312	176	182	0	0	149	161	183	199	233	233	139	139
337	N2	C	170	222	300	312	176	182	201	203	149	149	177	183	0	0	139	139

ID	Stage	Structure	Microsatellite loci															
			A02_1	A02_2	E12_1	E12_2	F03_1	F03_2	C08_1	C08_2	E02_1	E02_2	C02_1	C02_2	D09_1	D09_2	C09_1	C09_2
338	N2	C	0	0	300	300	172	182	203	203	149	161	173	173	197	209	139	139
339	N2	C	170	222	300	312	172	176	203	203	149	161	173	177	209	209	139	139
340	N2	C	192	198	300	312	172	182	203	203	157	161	177	199	197	233	131	159
341	N2	C	170	198	300	312	172	182	201	203	149	149	177	177	217	233	139	139
342	N2	C	170	198	300	312	172	202	203	203	157	161	177	183	209	213	139	139
343	N2	C	170	198	300	312	176	182	203	203	149	161	177	183	209	213	139	139
344	N2	C	192	198	300	312	172	182	201	203	157	161	185	199	209	209	131	131
345	N2	C	0	0	300	312	176	182	203	203	149	157	163	177	197	197	139	139
346	N2	C	192	198	300	312	172	176	201	203	149	149	177	183	209	209	139	139
347	N2	C	170	184	300	314	172	202	201	203	149	149	177	183	197	233	139	147
348	N2	C	0	0	0	0	172	176	203	203	0	0	177	177	209	209	159	159
349	N2	C	170	170	300	314	172	178	203	203	149	157	163	183	197	209	139	159
350	N2	C	170	170	300	314	0	0	203	203	149	161	0	0	197	217	139	139
351	N2	C	198	198	300	312	172	182	203	203	149	149	177	183	197	197	139	139
352	N2	C	170	170	300	312	172	176	201	203	149	149	177	183	197	233	139	159
353	N2	C	170	170	300	312	172	176	203	203	149	149	173	183	197	209	139	159
354	N2	C	170	170	300	312	172	176	203	203	157	161	173	177	197	209	139	139
355	N2	C	182	192	300	312	172	176	203	203	149	157	175	185	205	217	139	139
356	N2	C	170	222	300	312	202	202	203	203	149	161	183	199	209	209	131	147
357	N3	C	170	222	300	300	172	182	203	203	149	149	177	183	197	233	131	139
358	N3	C	170	222	300	308	172	176	203	203	157	161	177	199	197	233	131	139
359	N3	C	190	190	300	314	176	182	203	203	149	157	177	183	197	209	159	159
360	N3	C	190	198	300	312	172	178	203	203	149	157	177	183	197	209	139	139
361	N4	C	170	184	312	312	172	172	201	203	149	157	177	177	209	209	139	139
363	N5	C	192	192	300	300	172	176	203	203	149	161	183	199	197	217	131	139
364	N1	C	170	198	312	312	172	176	203	203	149	157	183	199	209	233	139	139
365	N2	C	170	198	300	312	172	182	203	203	149	161	177	185	205	205	139	159
366	N3	C	170	198	300	312	172	182	201	203	161	165	177	183	209	209	131	131
367	N3	C	170	222	300	312	176	180	201	203	149	149	183	199	209	209	139	139
368	N4	C	192	192	312	314	172	176	201	201	149	157	177	177	197	209	139	139
369	N4	C	190	190	300	314	176	180	203	203	149	157	177	183	197	209	139	159
370	N4	C	0	0	300	308	172	176	203	203	149	157	173	183	197	217	139	139
371	N4	C	192	192	300	312	176	180	201	203	149	157	177	177	209	233	139	159
372	M	C	192	192	300	314	172	182	203	203	149	149	177	183	197	205	147	159
373	M	C	192	198	300	312	176	182	203	203	149	157	177	177	209	209	139	139
374	N5	C	170	198	300	312	172	182	203	203	157	161	177	199	209	233	139	139
375	H	C	170	222	300	300	172	180	201	203	149	157	173	177	197	209	139	139
376	H	C	170	222	300	300	172	180	201	203	157	157	177	177	197	209	139	139
377	M	C	170	170	300	312	172	202	201	203	149	161	173	199	197	217	131	147
378	M	C	198	198	300	312	172	182	203	203	149	161	177	183	197	197	139	139
379	N3	C	170	182	300	312	172	176	203	203	149	149	175	199	205	233	131	139
380	N5	C	192	192	300	312	176	182	203	203	149	157	175	177	197	209	139	139
381	H	C	170	192	300	312	172	176	203	203	149	157	173	177	209	209	139	159

Note: "0" denotes missing data or null alleles.
