# Supplementary material for: Coexistence of monogamy and polygyny in Triatoma infestans: fine-scale genealogical structure reveals complex social structures within domestic colonies in the Bolivian Chaco
Source: Mem Inst Oswaldo Cruz. 2026 Jun 15;121:e250355. doi: 10.1590/0074-02760250355 (PMC13268354; doi:10.1590/0074-02760250355)
Supplement: Peer review history

# Reviewer report: FIRST REVIEW ROUND - REVIEWERS' COMMENTS

## REVIEWER #1

Dear Editor,

Please receive my comments regarding manuscript MIOC-2025-0355.

The study addresses a relevant and interesting question, and the general conceptual framework is promising. However, in its current form the manuscript presents major methodological shortcomings and interpretative issues that substantially weaken the validity of the conclusions. Insufficient genetic resolution, lack of critical analytical details, and unsupported causal inferences between mating system and reproductive success limit the robustness of the results.

Below I point the main issues about the article:

1. Bibliography

The bibliography is very limited, comprising only 10 references, which is well below what is expected for a research article of this scope. Several citations referring to "recent work" or to the current infestation status in the Gran Chaco ecoregion are more than a decade old, and key studies addressing the genetic structure of *T. infestans* at a fine geographic scale are not cited. In addition, the reference Fronza et al. 2023 does not appear to exist and may have been generated artificially.

2. Data analysis

Although the general analytical approach is described, several essential details required for reproducibility and proper evaluation are missing.

First, the study relies on only eight microsatellite loci to analyze 381 individuals. For high-resolution pedigree reconstruction in a potentially inbred domestic colony, this number of loci may be insufficient to reliably discriminate among full-siblings, half-siblings, and more distant relatives. The authors should therefore report standard metrics such as exclusion power or probability of identity for the marker set used.

Second, while COLONY v2.0 is cited, the manuscript does not specify key model settings, including likelihood options (e.g. long vs. short runs) or assumed genotyping error rates (allelic dropout and false alleles). These parameters can strongly affect sibship reconstruction and, consequently, the inference of 42 family groups.

Finally, the finite growth rate (λ) is used as a proxy for fitness, but neither the formula nor the stage-structured matrix underlying its calculation is described. Without this information, the interpretation and comparability of λ values remain unclear.

3. Interpretation of the relationship between monogamy and "success"

The authors report that the two most abundant families (F1 and F2) were monogamous, whereas the remaining 40 minor families showed polygamous mating patterns. However, this pattern alone does not support a causal relationship between monogamy and reproductive success. The manuscript itself suggests that the dominance of F1 and F2 may reflect a founder effect, with these families being among the first colonizers. Under this scenario, their apparent success could be explained by priority effects and availability of mates, while later arrivals may experience increased competition and reduced reproductive opportunities.

A major limitation of the study is the use of microsatellites—neutral genetic markers—to infer adaptive success. Because these markers do not provide information on traits under selection or functional fitness, the analysis cannot disentangle historical demographic processes (such as founder effects or arrival order) from true natural selection favoring monogamy. Consequently, attributing the dominance of families F1 and F2 to a superior reproductive strategy, or characterizing minor families as "less adapted," remains speculative. Similarly, a high growth rate (λ) in a neutral lineage may simply reflect favorable initial conditions (e.g. access to a suitable refuge or blood source) rather than any underlying genetic advantage.

## REVIEWER #2

The manuscript entitled "Coexistence of Monogamy and Polygyny in *Triatoma infestans*: Fine-scale genealogical structure reveals complex social structures within domestic colonies in the Bolivian Chaco" presents an original approach exploring interesting ecological aspects on *Triatoma infestans* the main *T. cruzi* vector in the south of South America. The authors submitted the manuscript as a research article, however the background for this kind of manuscript is too superficial specially focusing a relevant vector as *T. infestans*.

Several recommendations are listed below:

According to the International Code of Zoological Nomenclature, the very first time a species name is cited in a manuscript it must be written in full and followed by its authors name and date.

The authors drives the introduction strictly related to *T. infestans*. In order to enrich this topic we recommend including a few more references framing *T. cruzi* vectors in general before starting with *T. infestans*:

Lent, H.; Wygodzinsky, P. Revision of the Triatominae (Hemiptera, Reduviidae), and their significance as vectors of Chagas' disease. Bull. Am. Mus. Nat. Hist. 1979, 163, 123–520.

Cleber Galvão, Triatomine Bugs: History, Control, and Citizen Surveillance Dis. Biol. Genet. Socioecol. 2025, 1(3), 8

Costa, J.; Dale, C.; Galvão, C.; Almeida, C.E.; Dujardin, J.P. Do the new triatomine species pose new challenges or strategies for monitoring Chagas disease? An overview from 1979–2021. Mem. Inst. Oswaldo Cruz 2021, 116, 1–10

The approach is based on microsatellite analyses and several papers have already been published using this tool to explorer population aspects of the triatomines. The authors do not make proper references on the importance of the molecular tools in the improvement of the knowledge of *T. cruzi* vectors. We suggest a few words on this matter in the introduction and recommend some of the references.

The term "domestic" is not technically the most appropriate since can be associated to genetic processes therefore we recommend changing it for "domiciliary".

In the M&M relevant information is missing and must be included. For example, the authors do not mention a word on the control actions history in the study site. How frequently the site is inspected for triatomines infestation and eventually sprayed (or not), please provide information on this matter; also, the authors do not mention based on what criteria the specimens were identified as *T. infestans*. Please explain the identification procedures including the major reference (Lent, H.; Wygodzinsky, P. Revision of the Triatominae (Hemiptera, Reduviidae), and their significance as vectors of Chagas' disease. Bull. Am. Mus. Nat. Hist. 1979, 163, 123–520.). Also, it is important to mention the geographical coordinates for the study area; the period of the year as well as the time spent during the field activities must be detailed. Finally, the total number of collected specimens, how many insects in each of the ecotopes (structure A, B and C) were recorded and the respective developmental phases are available in the microsatellite loci table. I suggest mentioning this data in the M & M to make reading easier.

As said before, the authors should bring more correlated references to enrich the manuscript, especially in the discussion showing some comparisons with previous papers using microsatellites even if the study was carried out focusing a different species:

Bezerra, C.M., Belisário, C.J., D'Ávilla Pessoa, G.C. et al. Microsatellite variation revealed panmictic pattern for *Triatoma brasiliensis* (Triatominae: Reduviidae) in rural northeastern Brazil: the control measures implications. BMC Genet 21, 92 (2020). https://doi.org/10.1186/s12863-020-00903-w

Panzera, F.; Cuadrado, Á.; Mora, P.; Palomeque, T.; Lorite, P.; Pita, S. Differential Spreading of Microsatellites in Holocentric Chromosomes of Chagas Disease Vectors: Genomic and Evolutionary Implications. Insects 2023, 14, 772. https://doi.org/10.3390/insects14090772

Belisário, C.J., Pessoa, G.C.D., Silva, E.M. et al. Genetic characterization of residual *Triatoma infestans* populations from Brazil by microsatellite. Genetica 145, 105–114 (2017). https://doi.org/10.1007/s10709-017-9949-y

Because the authors mention the effects of the possible stress caused by *T. cruzi* infection on the triatomines biology, some information about the natural infection rates on the studied area should be presented.

Final comments: i- background must be significantly improved; ii- relevant information in the M & M must be provided; iii- the discussion should be broader, including and discussing previous papers that used microsatellites. In the conclusions, findings should be presented as suggestive since the obtained results must be confirmed in other sites before the acceptance of the findings as a rule for *T. infestans* population distribution. According to the mentioned points, the reviewed study is in the scope of MIOC and has merits to be published in this relevant journal however, before the final acceptance of the manuscript, significant changes and corrections, as mentioned above, must be carried out.

## REVIEWER #3

The manuscript presents important information on the reproductive aspects of one of the main vector species for Chagas disease. The observed social and genetic structure allows for new paths to be forged by vector control programs (highlighting the need to consider all aspects of One Health). However, before being accepted for publication in MIOC, some issues need to be improved/clarified:

Line 1

Change "*Triatoma infestans*: Fine-scale" for "*Triatoma infestans*: fine-scale"

Line 20

Change "...were reconstructed using maximum likelihood analysis (COLONY)" for "were reconstructed using maximum likelihood analysis with the software COLONY v2.0"

Line 30

Do not repeat words from the title, such as *Triatoma infestans*.

Lines 33-35 - Include reference(s).

Lines 38 and 39 - Include reference(s).

Lines 40-42 - Include reference(s).

Line 43 – "If a domestic "colonia"..." - why do the authors use the term "colonia" instead of "colony"?

Lines 45 and 46 - Include reference(s).

Lines 47-51 – I suggest that the authors cite articles that reported polyandry in Triatominae (for example, Melgar et al., 2007 and, mainly, Manrique and Lazzari, 1994 who described the phenomenon for *T. infestans*)

Melgar S, Chávez JJ, Landaverde P, Herrera F, Rodas A, Enríquez E, Dorn P, Monroy C. The number of families of *Triatoma dimidiata* in a Guatemalan house. Mem Inst Oswaldo Cruz. 2007 May;102(2):221-3. doi: 10.1590/s0074-02762007005000001. PMID: 17426889.

Manrique G. Lazzari C. R. 1994. Sexual behaviour and stridulation during mating in *Triatoma infestans* (Hemiptera: Reduviidae). Mem. Inst. Oswaldo Cruz 89: 629–633.

Lines 86 and 87

"These families originated from a minimum of 21 founder progenitors (11 females and 10 males)" - How did the authors obtain this information? Specify.

Line 105 - The discussion needs to be supplemented with information from the literature on the reproductive aspects of *T. infestans*. I suggest that the authors conduct an in-depth search on the subject. A quick search allowed me to obtain the following references:

MANRIQUE G. LAZZARI C. R. 1994. Sexual behaviour and stridulation during mating in *Triatoma infestans* (Hemiptera: Reduviidae). Mem. Inst. Oswaldo Cruz 89: 629–633.

HERNÁNDEZ, M. L., et al. 2018. Does nutrition influence sexual dimorphism in *Triatoma infestans* (Hemiptera: Reduviidae) of natural habitats?. Revista De La Sociedad Entomológica Argentina, 77(1). Retrieved from https://www.biotaxa.org/RSEA/article/view/37708

ALEVI, KCC, et al. 2018. Reproductive aspects of Chagas disease vectors (Hemiptera, Triatominae) with anatomical teratologies. ACTA TROPICA, v. 185, p. 251-254, 2018.

DE OLIVEIRA, A.B.B., et al. 2020. Parasite × vector relationship in Chagas disease: does *Trypanosoma cruzi* (Chagas, 1909) infection affect the spermatogenesis of *Triatoma infestans* (Klug, 1834)?. Parasitol Res 119, 3517–3522 (2020). https://doi.org/10.1007/s00436-020-06788-z

GIOJALAS, L. C. AND S. CATALA. 1993. Changes in Male *Triatoma infestans* Reproductive Efficiency Caused by a Suboptimal Temperature. Journal of Insect Physiology 39(4): 297-302.

MONTEIRO FA, et al. 1999. Mitochondrial DNA variation of *Triatoma infestans* populations and its implication on the specific status of *T. melanosoma*. Mem Inst Oswaldo Cruz. 1999;94 Suppl 1:229-38. doi: 10.1590/s0074-02761999000700037.

ANTUNES, C et al. 2020. Sexual Choice in Males of the *Triatoma brasiliensis* Complex: A Matter of Maintenance of the Species or Genetic Variability?. THE OPEN PARASITOLOGY JOURNAL, v. 8, p. 1-9, 2020.

Furthermore, I find it interesting that the authors discuss the microsatellite results in comparison to those published for other triatomine species.

Line 116

Change "Lobbia et al. (2024)" for "Lobbia et al. [7]"

Line 132 - put in italics

Line 154 - all references need to be reviewed and scientific names need to be in italics.

Figures - The text in figures 3 and 4 needs to be in English.

## REVIEWER #4

This is a well written short manuscript that describes the genealogical relationships, mating behavior (monogamous vs polygamous), and demography of 381 *Triatoma infestans* from the Bolivian Chaco. Insects were collected from three neighboring structures (2 bedrooms and a chicken coop), and molecularly genotyped using eight published microsatellite loci. Kinship and mating systems were determined via maximum likelihood analyses using the COLONY software. The main findings are that the 42 families identified and their social structure are shaped by two different reproductive strategies: monogamy and polygyny. Finally, I believe this manuscript brings original contribution for the often overlooked field of study on the genealogy and kinship of insect vectors.

Major comments

The main issue I have with this manuscript is that because it is so short, it seems that many details, explanations, and important references were left out. For example, there is no information on how microsatellite alleles were scored. What softwares were used? The reproductive biology/behavior of *T. infestans* could have been addressed in more detail as well. Are there specimens (both male and female) that are naturally prone to be more promiscuous? What is known about this behavior? Is this genetically determined?

Shouldn't there be, perhaps in Section 2.3, a word or two on parameters chosen/assumptions made in the analyses? For example, was it assumed that all scored alleles are Identical by Descent?

Relevant references on the epidemiological importance of *T. infestans* in the Chaco region were not included. Only 3 refs are cited in the Discussion section!

Minor comments

Line 16: the term "genetic structure" is mentioned here but seems not to have been discussed in the manuscript.

Line 17: it says "rural community" but only 2 bedrooms and a chicken coop were analyzed.

Line 43: I suggest changing "colonia" to colony.

Line 63: When was the fieldwork carried out? Please provide more detail: man/hour, tweezers, etc.

Lines 63-65: Are these just isolated bedrooms? Not huts? Please explain.

Line 67: How many specimens per structure? Some information is given in Figure 4 but it looks like only 3 families were considered.

Line 88: "Stark" is an unusual choice of work for a scientific paper.

Line 110: It was not obvious to me where the "paradox" resides.

Line 113: "founder effect" advantage. If the domestic niche was colonized by a single insect couple wouldn't they have to be monogamous? Please explain.

Line 126: Please better explain the meaning of "competitive release".

Line 203 reads: "(Bedroom A, Chicken Coop B, Bedroom C)". It seems odd to present the structures like this. "(Bedroom A, Bedroom B, and Chicken Coop)" would make more sense. I suggest changing this in the entire manuscript.

Table 1: Please correct the number of females that mated with Male 1. It should be 3 instead of 1. In addition, how to explain the fact that Male 322 had 6 offspring each with a different female?

Figure 3: Figure legend should read "Families F1 and F4 (predominantly monogamous origin)…", instead of "Families F1 and F2…". This is a very important point. Please check and correct this discrepancy throughout.

Figure 4 is in Spanish.

## AUTHORS' RESPONSE TO THE REVIEWERS

Response to Reviewers / Rebuttal Letter

Dear Dr. Ademir de Jesus Martins Jr.

Handling Editor, Memórias do Instituto Oswaldo Cruz

We would like to thank the Editor and the Reviewers for their rigorous and constructive evaluation of our manuscript (MIOC-2025-0355). Their insightful comments have been instrumental in improving the quality, clarity, and scientific depth of our work.

We have carefully addressed all concerns, expanding our methodological descriptions, updating the bibliography, and incorporating a deeper population genetics analysis (including bottleneck and expansion tests) to support our conclusions regarding the mating systems and demographic success of the analyzed families.

Below, we provide a point-by-point response to each reviewer's comments. Changes in the manuscript are highlighted in the revised version.

**Reviewer 1**

**1. Bibliography is limited (10 references), outdated, and the Fronza et al. 2023 reference appears incorrect.**

Response: We entirely agree. The bibliography has been comprehensively expanded and updated from 10 to 33 references. We have included recent key studies addressing the genetic structure of *T. infestans* at a fine geographic scale, the importance of molecular tools, and behavioral/reproductive aspects. The incorrect reference has been rectified; it now properly cites Gürtler et al., 2023 (PLoS Negl Trop Dis).

**2. Data analysis: Eight loci may be insufficient. Missing COLONY v2.0 settings. Missing formula/matrix for finite growth rate (λ).**

Response: We have significantly strengthened the Materials and Methods section to address these valid points:

• Resolution: We added the standard metrics. The loci panel showed a mean H_E_ of 0.675 and provided a Combined Exclusion Probability > 0.999, sufficient for high-confidence sibship discrimination (Section 2.2).

• COLONY settings: We specified the parameters: a full-likelihood method, medium run length, high likelihood precision, and an assumed genotyping error rate of 0.01 (Section 2.3).

• Growth rate (λ): We clarified that λ was derived from a stage-structured matrix based on transition probabilities between specific nymphal stages (N_t_ to N_t+1_) to obtain the geometric mean of growth (Section 2.3).

**3. Interpretation of the relationship between monogamy and "success": Neutral markers cannot infer adaptive success; it could just be a founder effect.**

Response: We deeply appreciate this insightful comment. To address it, we incorporated a new demographic/genetic analysis (Heterozygosity excess/deficiency tests) into the manuscript. As shown in the new Section 3.3 ("Divergent Demographic Histories") and Figure 4, we demonstrate that the two dominant families have distinct histories: Family F1 shows a signature of recent population expansion (Heterozygosity deficit), while Family F2 shows a signature of a recent genetic bottleneck (Heterozygosity excess). We have rewritten Section 4.1 ("Mating Systems and the Founder Effect Paradox") to reflect that our neutral markers show a coexistence of expansion and recovery strategies (resilience), rather than claiming a direct adaptive genetic advantage of monogamy.

**Reviewer 2**

**1. The very first time a species name is cited it must be written in full and followed by its authors name and date.**

Response: Corrected. It now reads *Triatoma infestans* (Klug, 1834) in the Introduction.

**2. Enrich the introduction framing T. cruzi vectors in general before starting with T. infestans. Add words on the importance of molecular tools and specific references.**

Response: We have broadened the introduction to frame Chagas vectors globally before narrowing it to *T. infestans* in the Gran Chaco. We also explicitly highlighted how microsatellite markers have revolutionized our understanding of vector population dynamics, incorporating the suggested reference (Bezerra et al., 2020) (Section 1).

**3. Change the term "domestic" for "domiciliary".**

Response: We have replaced "domestic" with "domiciliary" throughout the methodological and descriptive sections where appropriate.

**4. M&M missing information: control actions history, criteria for specimen identification (Lent & Wygodzinsky), geographical coordinates, time spent in field activities, total specimens per ecotope.**

Response: All requested details have been incorporated into Section 2.1. We included the geographical coordinates, specified the "man-hour effort method (1 hour per structure)", and explicitly cited Lent & Wygodzinsky (1979) for taxonomic identification. The demographic breakdown by ecotope was summarized to improve readability. Regarding control history, the area is under standard irregular programmatic surveillance, which correlates with the recent "bottleneck" genetic signature we found and now discuss.

**5. Some information about the natural infection rates on the studied area should be presented.**

Response: While we agree this is epidemiologically relevant, parasite detection was not performed on this specific sample set. However, we have included literature (Lobbia et al., 2024) in the discussion to address how *T. cruzi* infection can act as an environmental stressor altering reproductive trade-offs.

**Reviewer 3**

**1. Minor text corrections (Capitalization, COLONY v2.0 software, "colonia" vs "colony", Italics).**

Response: All suggested typographical corrections, including the proper use of italics for scientific names and the translation of Spanish terms in figures ("colonia" to "colony"), have been fixed throughout the text and images.

**2. Include references in several lines. Cite articles that reported polyandry (Manrique and Lazzari 1994).**

Response: We have thoroughly referenced the manuscript. The foundational work by Manrique & Lazzari (1994) on mating behavior and stridulation is now cited to contextualize the reproductive strategies observed.

**3. "These families originated from a minimum of 21 founder progenitors" - How did the authors obtain this information? Specify.**

Response: We clarified in the text that this was inferred directly by the Maximum Likelihood sibship reconstruction algorithm executed by COLONY v2.0, which computes the most likely number of parents required to generate the observed offspring genotypes.

**4. The discussion needs to be supplemented with information from the literature on the reproductive aspects of T. infestans.**

Response: We have significantly enriched the discussion (Section 4.1) by addressing the "paradox of polygamy" in the domiciliary environment, incorporating references on reproductive biology, biological costs, and physiological trade-offs (e.g., Lobbia et al., 2024; Sierwald et al., 2024).

**Reviewer 4**

**1. No information on how microsatellite alleles were scored. What softwares were used? Parameters chosen/assumptions made?**

Response: We have substantially expanded Sections 2.2 and 2.3. We now explicitly state the use of an ABI PRISM 377 sequencer, Geneious Pro v5.6 for allele calling, Micro-Checker for error assessment, GenAlEx, and GenePop. For COLONY, we added the assumptions made: polygamous model, full-likelihood, and an allelic dropout rate of 0.01.

**2. Relevant references on the epidemiological importance of T. infestans in the Chaco region were not included.**

Response: We have expanded our reference list to 33 citations, incorporating key epidemiological and population genetics literature relevant to the Chaco region and vector control implications (e.g., Gürtler et al., 2023; Gaspe & Gurtler, 2013; Marcet et al., 2008; Lardeux, 2013).

**3. Line 63: Fieldwork details (man/hour, tweezers). Lines 63-65: Isolated bedrooms vs huts?**

Response: We added the requested fieldwork details (1 hour per structure, use of tweezers). We also clarified that the sampled structures are individual units within a single precarious household compound (illustrated in the new Figure 1).

**4. Line 110: "Paradox" explanation. Line 113: "founder effect". Line 126: "competitive release".**

Response: We have clarified these concepts in the Discussion. The "paradox" is explained by contrasting the theoretical advantage of polyandry (genetic diversity) with our finding that the most numerically successful families were monogamous. The "competitive release" refers to minor lineages rapidly expanding once the dominant families are eliminated by focal spraying. The "founder effect" was addressed by incorporating the new genetic bottleneck vs. expansion analysis.

**5. Table 1 discrepancies and Figure 3 legend error (F1 and F4).**

Response: Table 1 has been completely redesigned to be clearer, summarizing the findings into "Dominant" and "Minor" lineages, removing the confusing individual progenitor rows. The typo in the Figure 3 legend (F4 instead of F2) has been corrected. All figures are now fully in English.

# Reviewer report: REVIEWERS' COMMENTS

## REVIEWER #1

The manuscript was greatly improved; the authors answered properly the corrections and suggestions recommended by the referees however, a few points still require authors consideration.

In the introduction, the authors presented a brief paragraph on *T. cruzi* vectors, as recommended, mentioning the most important review (Lent and Wygodzinsky, 1979) together with other relevant papers not updated. It would be important including recently published manuscripts as previously recommended.

Cleber Galvão, Triatomine Bugs: History, Control, and Citizen Surveillance Dis. Biol. Genet. Socioecol. 2025, 1(3), 8

Costa, J.; Dale, C.; Galvão, C.; Almeida, C.E.; Dujardin, J.P. Do the new triatomine species pose new challenges or strategies for monitoring Chagas disease? An overview from 1979–2021. Mem. Inst. Oswaldo Cruz 2021, 116, 1–10

Please check in the results the term "sib family" line 120

## REVIEWER #2

Dear Editor,

My queries have been properly addressed. I now find this manuscript acceptable for publication in the Memorias journal.

## REVIEWER #3

The authors incorporated most of the suggestions presented in the first round of revision. Therefore, I consider this new version suitable for publication in MIOC.

## REVIEWER #4

No comments.
